# Association Between Family Planning and Early Childhood Development: Secondary Analysis of the 2018 Turkey Demographic and Health Surveys

**DOI:** 10.3390/children12020220

**Published:** 2025-02-12

**Authors:** Gamze Gezgen Kesen, Sıddıka Songül Yalçın

**Affiliations:** 1 Department of Pediatrics, Etimesgut State Hospital, Ankara 06790, Türkiye; 2 Department of Social Pediatrics, Institute of Child Health, Hacettepe University, Ankara 06230, Türkiye; siyalcin@hacettepe.edu.tr; 3 Department of Pediatrics, Hacettepe University Faculty of Medicine, Ankara 06230, Türkiye

**Keywords:** early childhood development index, demographic and health survey, family planning

## Abstract

**Background and Objectives:** Early childhood development (ECD) significantly influences long-term academic and social outcomes. Family planning (FP) can affect ECD by altering family dynamics and resource allocation. We aimed to investigate the association between the unmet need for family planning (UMNFP) and early childhood development (ECD), as well as family child care practices in Türkiye. **Materials and Methods:** We included mothers with children under 5 years from the “2018 Turkey Demographic and Health Surveys” (TDHS). Dependent variables included the ECD index, child care practices. Independent variables included FP needs, child–family characteristics. The study applied complex sample analysis. **Results:** The UMNFP was not associated with poorer child care practices; however, the availability of multiple types of toys or books was associated with the presence of UMNFP. Children from families with UMNFP had engaged in a smaller number of activities compared to their peers. However, on developmental assessments, these children did not perform differently than the comparison group. Factors such as maternal education and socioeconomic status also significantly moderated these effects, highlighting the complex interplay between FP and ECD. **Conclusions:** UMNFP is not associated with adverse child care practices but is related inversely to some educational activities and engagement in Türkiye, though no direct relationship with early childhood development (ECD) outcomes was identified. Expanding access to FP services and addressing socioeconomic disparities have the potential to improve child care practices, thereby contributing to more equitable developmental outcomes nationwide.

## 1. Introduction

### 1.1. Early Childhood Development

Early childhood development (ECD) is the first 8 years of life when growth and development are most accelerated in humans [1,2]. The rapid growth and development during this period significantly amplify the long-term impact of any damage or interventions [1,3]. As a result, such influences tend to have more profound and lasting consequences. A study by Garca et al. demonstrated the substantial long-term economic benefits of early childhood education programs, yielding an internal rate of return of 13.7% and a benefit–cost ratio of 7.3, emphasizing the social and economic efficacy [1].

ECD is influenced by various biopsychosocial factors, with numerous studies highlighting strong correlations between poverty, socioeconomic status, and child development [4,5,6,7,8]. Parental education, especially that of mothers, is a critical determinant of ECD [9,10,11]. Dysfunctional household environments have also been shown to adversely affect ECD [11]. Promoting family and community well-being significantly enhances child development during the early years and beyond [2,12]. Recent initiatives such as “Education for All”, the Millennium Development Goals (MDG), and the Sustainable Development Goals (SDG) have contributed to reducing malnutrition, illiteracy, infant mortality, stunting, and related challenges also aimed at enhancing child development [13,14].

Parenting practices and the quality of the home environment are key determinants of ECD outcomes, with significant long-term implications for children [12,15,16,17]. Interventions promoting responsive caregiving and early learning have proven to be effective in mitigating risk factors such as poverty and insufficient parenting skills in both high- and low-income settings [2,12,18]. Recent studies have highlighted the positive impact of fathers’ involvement in childcare on child development across diverse socioeconomic contexts [17,19,20,21].

### 1.2. Unmet Need for Family Planning and Child Development

The unmet need for family planning (UMNFP) refers to women who are fertile, sexually active, and wish to delay or limit childbirth but are not using contraception. UMNFP is a major public health issue with significant adverse implications for maternal and child health [22,23]. Risky fertility behaviors, such as short inter-pregnancy intervals after live births, are strongly linked to higher perinatal mortality [24]. In low- and middle-income countries, delaying first births and extending inter-pregnancy intervals substantially reduce stunting prevalence and improve child growth and development [25]. Universal access to family planning (FP) is a fundamental human right, crucial for promoting gender equality, empowering women, reducing poverty, and achieving Universal Health Coverage as outlined in the Sustainable Development Goals (SDGs) [26]. Contributing factors often include poverty, limited contraceptive options, unemployment, low education levels, restricted access to family planning services, cultural or religious beliefs, concerns about contraceptive side effects or health risks, inadequate service quality, gender-based barriers, and urban–rural disparities and both the definition and calculation of UMNFP [27,28,29].

In Türkiye, UMNFP increased significantly from 5.9% in 2013 to 11.6% in 2018, according to data from the Turkey Demographic and Health Surveys (TDHS) [30,31]. In 2018, 11.6% of women in Türkiye had an UMNFP, with 4.0% desiring to space births and 7.6% seeking to limit fertility [32].

For the theoretical framework, this study is grounded in the Resource Dilution Theory, which posits that as family size increases, resources allocated per child diminish, potentially leading to a decline in the quality of care [33,34]. This is complemented by Bowlby’s Attachment Theory, which emphasizes the critical role of secure early attachments in fostering a child’s socio-emotional and psychological well-being [35]. UMNFP may strain parental resources, reducing emotional availability and compromising the quality of attachment. Together, these frameworks underscore the intricate relationships between family size, resource allocation, emotional bonding, and developmental outcomes, forming the basis for investigating the potential long-term impacts of UMNFP on ECD [36]. These theories jointly provide the foundation for hypothesizing that UMNFP may compromise both material and emotional caregiving resources, ultimately affecting developmental outcomes in children.

Despite extensive research on ECD, there is a lack of comprehensive studies exploring the interplay between UMNFP, childcare practices, and ECD outcomes, particularly within the sociocultural context of Türkiye. To address this gap, this study aims to examine the relationship between UMNFP and childcare practices, as well as the relationship between ECD and UMNFP, taking childcare practices into account with secondary analysis of the 2018 TDHS.

This study hypothesizes that the presence of UMNFP in a family is associated with less optimal childcare practices, including reduced time spent on early stimulation activities such as storytelling, playing, and learning. Furthermore, it is proposed that children in families with UMNFP exhibit delayed ECD indicators compared to their peers. Additionally, the association between UMNFP, childcare practices, and ECD outcomes is expected to vary based on sociodemographic factors, such as maternal education level, household income, and urban or rural residence.

The findings of this study have the potential to inform public health policies by highlighting the impact of unintended pregnancies on early childhood outcomes. This could guide the development of targeted interventions aimed at improving childcare practices and early stimulation activities aimed at achieving SDGs.

## 2. Materials and Methods

Data are obtained from the 2018 TDHS [30]. TDHS data are nationally representative household surveys with weighted, multistage, stratified cluster sampling collected by face-to-face interviews. Sample selection is made by using the database from the Ministry of Internal Affairs. The dataset included child development, childcare practices, and family planning across the nation.

Inclusion criteria for the secondary analysis were mothers having children under 5 years old. Children born from multiple pregnancies and preterm births (born before 32 weeks) were excluded from the study due to their higher risk of developmental delays compared to the general population. These exclusions were made to ensure the sample focused on typical developmental trajectories and to minimize confounding factors.

Additionally, children not living with their biological mothers were excluded because UMNFP was assessed specifically with the mothers, and the ECDI (Early Childhood Development Index) was based on responses provided by the mothers. Data regarding deceased children were also excluded, as the survey collected comprehensive information about all children of the participating mothers, and deceased children could not contribute to the study’s developmental outcomes.

**Figure 1:**
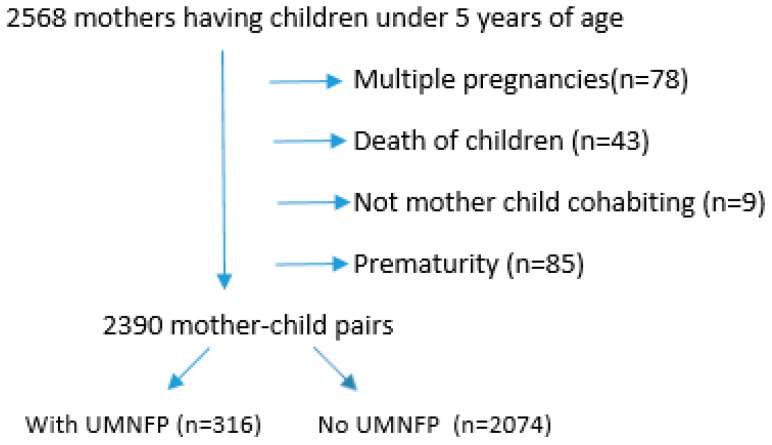
Flow chart of included mother–child pairs.

Data from 2568 children in the TDHS-2018 were evaluated and 2390 mothers having children under 5 years were included in the study (Figure 1)**.** This secondary analysis covered 93% of the sample. There were 1480 children between the ages of 2–4 and 1013 children between the ages of 3–4.

### 2.1. Variables

The families’ household characteristics [region, residence (urban, rural), household size, wealth index (poorer, middle, richer)], parental characteristics (mother’s and father’s age at birth, education level, mother’s employment status), child’s characteristics (age, sex, preceding birth interval), presence of UMNFP, mother’s pregnancy status at the time of survey, if she wanted her last child, children’s antenatal and perinatal characteristics [size at birth according to mother, gestational duration, number of antenatal care received in the first trimester] were taken as independent variables. All ages were determined based on exact dates of birth whenever possible, verified through the examination of identification cards when available [30].

The primary dependent variable was “ECD index (ECDI) score for children between 36 and 48 months”.

The secondary dependent variables were “activity engagement status for children aged 24–59 months of age”, “inadequate care under 5 years of age”, and “educational items at home for children under 5 years of age”. These variables were utilized in specific age groups because the DHS data were collected within these limits only (for example activity engagement status information was only obtained from the children between 24 and 59 months of age).

### 2.2. Definitions

In the 2018 TNSA, the UMNFP rate is calculated as the ratio of the total number of women who are not pregnant, not in the amenorrheic period following delivery, capable of childbearing, wish to delay their next birth for two or more years, or wish to end childbearing but are not using any method, women who have had an untimely or unwanted current pregnancy and those in the amenorrheic period post-delivery who did not want their births in the last two years to the married female group of 15–49 years [30,37].

Inadequate care was defined as “leaving the child under age 5 alone or in the care of another child younger than age 10 for more than 1 h at least once in the last week prior to the survey”, and collected for children under the age of 5 [30,38,39].

Mothers were asked about the number of children’s books and types of playthings available at home for children under 5 years old. When evaluating children’s playthings, the diversity of toy types was assessed. A greater variety of toy types contributes to children’s development by fostering their creativity. Learning or educational materials, items, and toys were categorized as follows: “having two or more different types of playthings at home”, and “having three or more children’s books or picture books” [30,38,39].

Additionally, mothers were asked whether their children aged between 24 and 59 months had engaged in any activities with a person aged 15 years or older, mother, or father in the last three days that promote learning and school readiness. A mother or father engaging in four or more activities with the child during the last three days was considered appropriate for children aged 24–59 months [30,38,39].

The Early Childhood Development Index (ECDI), developed by UNICEF, is a tool designed to assess the developmental progress of children aged 36–59 months [30,38,39,40]. It provides a comprehensive measure of a child’s overall development across key domains critical to early childhood, including literacy–numeracy, physical development, socio-emotional development, and learning. The ECDI is particularly valuable in low- and middle-income countries, where standardized developmental assessments may be scarce or inaccessible. A child is considered “on track” if they exhibit typical development in at least three of these domains.

*Literacy–Numeracy Domain: This domain evaluates whether a child can “recognize at least 10 letters of the alphabet”, “read at least four simple words”, and “identify and understand numbers from 1 to 10”. A child is classified as on track if they meet at least two out of these three criteria.

*Physical Development Domain: This domain assesses the child’s ability to “pick up small objects using two fingers” and whether the child “is too sick to play frequently”. Achieving at least one out of these two indicators qualifies as being on track.

*Learning Domain: This domain examines whether the child can “follow simple instructions” and “complete a task independently”. A child is considered on track if they meet at least two out of three criteria.

*Socio-Emotional Development Domain: This domain explores the child’s ability to “get along well with other children”, their likelihood to “bite, kick, or hit others”, and whether they “become easily distracted”. A child is on track if they score positively on at least two out of these three indicators.

### 2.3. Data Analysis

Statistical analyses were conducted using IBM SPSS Statistics version 22 (International Business Machines Corporation, Armonk, NY, USA) and STATA version 13.0 (StataCorp LP, College Station, TX, USA).

To address potential biases related to differences in sample sizes, weighting factors were applied. These factors were calculated using the formula 1/(a × nc/nT), where “a” represents the total number of surveys, “nc” denotes the number of respondents in survey “c”, and “nT” is the aggregate number of respondents across all surveys. The demographic and clinical characteristics of child and their family were presented as weighted samples (n, %). Analyses accounted for the complex survey design, incorporating CSPLAN files to ensure accurate representation. Using the SPSS database, CSPLAN was created in the Complex Samples module by utilizing sample weights, stratification, and cluster information [37]. To evaluate changes in the frequencies of dependent variables within independent variable subgroups, complex sample crosstabulations were performed. Chi-square tests, along with adjusted residuals, were used to explore interactions within the weighted sample.

Multivariable logistic regression models were developed for each dependent variable. Independent variables with a *p*-value < 0.20 in the initial chi-square tests were included as candidate predictors in the regression analyses.

A significance threshold of *p* < 0.05 was applied to identify statistically significant results, corresponding to a 95% confidence level.

## 3. Results

### 3.1. Inadequate Care

Among 2390 children under 5 years of age, 6.6% were left alone or under the care of a child under 10 years of age which was considered inadequate care. A total of 3.4% were left alone and 4.1% stayed with a young child in the last week before the survey (Table 1).

Inadequate care prevalence did not change significantly according to the region. Living in rural areas, residing in crowded households, being part of poorer households, having a father with lower education levels, and being three years old were associated with a higher prevalence of inadequate care than their counterparts (*p* < 0.05, Table 1).

There was a significant association between the total number of children and inadequate care. The first-born child had the lowest percentage of inadequate care, while the highest percentage was observed among the fourth-born and later children who were born with a birth interval of 24 months or more. This situation was not statistically significant for cases of being left entirely alone but remained significant for instances where a child was left with another child. Contrarily, mothers being pregnant at the time of the survey was inversely related to staying with a young child (*p* = 0.005, Table 1).

An increase in the number of children the mother had given birth was associated with the practice of child-to-child caregiving and inadequate care (*p* < 0.001, Table 1). Interestingly, if the child was born preterm, the likelihood of being left alone was lower than counterparts (*p* = 0.044, Table 1). 

### 3.2. Children’s Books and Playthings

Among a total of 2390 children under the age of five, 76.6% had access to two or more types of playthings (Table 1). The presence of two or more types of toys at home was significantly associated with living in urban areas, in the middle and western part of Türkiye, having less than five people in households, higher wealth index, higher levels of maternal and paternal education, maternal employment, three or less children in the family, being a firstborn child (*p* < 0.05, Table 1). Contrarily, children with smaller birth sizes, and children residing in the east region, having birth order ≥4, and having a mother with UMNFP were less likely to have access to at least two types of playthings at home than counterparts (*p* < 0.05, Table 1).

Out of 2390 children, only 716 (29.9%) had more than three children’s books at home. Having three or more children’s books at home was positively associated with living in urban areas, residing in less crowded households, higher household wealth index, advanced maternal and paternal age, higher maternal education levels, and presence of parental employment, fewer number of children in the family, and being a firstborn child (*p* < 0.05, Table 1). The presence of UMNFP was negatively associated with the presence of three or more books at home (*p* < 0.001). The likelihood of having more than three books was observed to increase with age, significantly (*p* < 0.001, Table 1).

### 3.3. Support for Learning: Activity Engagement

Among children aged 24–59 months (n = 1480), 66.0% had participated in four or more activities with an adult household member (aged 15 or older) in the three days preceding the survey. 49.8% engaged in activities with their mother, while 15.7% participated in activities with their father (Table 2).

Living in western Türkiye, residing in urban areas, belonging to wealthier households, having a household size of fewer than five members, and higher levels of maternal and paternal education and presence of maternal employment were all positively associated with children engaging in four or more activities with any adult (mother, father or household member) (*p* < 0.05, Table 2). Conversely, living in eastern Türkiye, younger maternal or paternal age at the time of the child’s birth, being the fourth or more child regardless of the precedence status, the presence of UMNFP, and the mother being pregnant at the time of the survey—potentially indicative of UMNFP—were inversely associated with children engaging in fewer than four activities with any adult (*p* < 0.05). Children of mothers who did not want their last children were less likely to engage in four or more activities with their mothers or fathers (*p* < 0.05) (Table 2). If the mother is pregnant at the time of the survey, the likelihood of engaging in an activity with any adult household member decreased and that decrease was statistically significant (*p* = 0.027) (Table 2). Father’s age at birth also had a statistically significant relationship with engaging in activities with the father, especially in the 25–34 year group (Table 2) (*p* < 0.05), whereas mother’s age was associated with engaging in an activity with any adult (*p* = 0.014) (Table 2).

### 3.4. Early Childhood Development Index (ECDI)

Overall, 1013 children between the ages 36 and 59 months were evaluated. Among a total of 1013 children, 74.3% were identified as having normal ECDI scores. Specifically, 14.6% of the children were on track in the literacy–numeracy section, 98.4% were successful in the physical development domain, 96% demonstrated on track development in the learning domain, and 74% were successful in the socio-emotional domain (Table 3). Due to the limited number of children in the subgroups of the physical and learning domains, further analyses were not conducted on these areas.

Our analysis revealed that being on track in the literary-numeracy domain of the ECDI was positively associated with living outside the eastern region of Türkiye, residing in urban areas, and living in higher wealth index families. Other positively related significant factors included living in households with fewer than five members, higher parental education levels, having a mother who reported wanting her last child, being a firstborn child (*p* < 0.05, Table 3). Additionally, children who were 4 years old, had three or more children’s books at home, and engaged in four or more learning and school readiness activities with an adult in the past three days (regardless of whether the adult was the mother, father, or another adult) were more likely to be on track in the reading domain (*p* < 0.05).

Being on track in the socio-emotional domain of the ECDI was strongly linked to living in households with fewer than five members, belonging to middle or richer wealth index households, having a 25 years or older father at the time of birth, higher maternal education levels, presence of maternal employment, being a female child, having three or more children’s books at home, and engaging in four or more educational activities with any adult in the past three days (*p* < 0.05, Table 3).

Regarding the total ECDI score, being on track was associated with living in the western and middle region, residing in a household with fewer than five members, and households in the middle or richer wealth indexes, having a father aged over 35 at the time of birth, higher parental education levels, presence of maternal employment, being older in age, being a female child, the mother not being pregnant at the time of the survey, having three or more children’s books at home, having two or more types of toys at home, and participating in more than four activities with any adult (*p* < 0.05, Table 3).

### 3.5. Further Analysis: Inadequate Care and Educational Materials

The regression analysis demonstrated significant regional disparities, with children in eastern regions having the lowest odds of owning three or more children’s books (AOR = 0.48, 95% CI: 0.26–0.90, Table 4).

Socio–economic status emerged as a critical factor, with children from a poorer wealth index had a lower chance of owning two or more types of toys and three or more children’s books indicated by odds ratios of 0.58 and 0.16, respectively (95% CI: 0.40–0.83 and 95% CI 0.11–0.23, respectively). (Table 4). Also, children living in families with less than five people had 1.42 times more likely to have three or more children’s books at home (95% CI: 0.26–0.90) (Table 4).

Maternal characteristics also played a crucial role, mothers aged 25 or older were nearly two times more likely to have households with more books compared to mothers younger than 25 years [AOR = 1.72 for 25–34 years (95% CI: 1.25–2.36) and AOR = 1.81 (95%CI: 1.09–2.99)]. Also, increased maternal education levels were associated with a 2.24-fold increased likelihood of having three or more children’s books at home (95% CI: 1.70–2.96) (Table 4). Households with working mothers showed also better educational resources, with 2.23 times higher odds of three or more books (95% CI: 1.67–2.99) (Table 4). The presence of UMNFP was also associated with less likelihood of owning three or more children’s books at home compared to the counterparts (AOR:0.52 95% CI: 0.35–0.78) (Table 4). Increased paternal education level was also an important factor that increased the likelihood of owning three or more books by 1.66 times (95% CI: 1.21–2.28) (Table 4).

Characteristics that have a relationship with insufficient caregiving were generally observed to be related to child-specific variables. It was found that children with an interval of more than 24 months from their preceding sibling had an increased risk of receiving insufficient care, with the risk being 1.57 times higher for second and third children born after 24 months compared to firstborns, and 2.15 times higher for fourth and subsequent children born after 24 months (95% CI: 1.00–2.46 and 1.19–3.91, respectively). Also, the odds of 3 year old children receiving insufficient care was 2.40 times higher compared to 0 year olds (95% CI: 1.37–4.21). This likelihood was 1.88 times in 2 year olds and 1.85 times in 4 year olds compared to 0 year old group (95% CI: 1.05–3.37 and 1.04–3.27, respectively).

### 3.6. Further Analysis: Educational Activities with Adults, Mother or Father

Regional differences were also present in the logistic regression analysis for educational activity engagement statuses. Children from the middle region of Türkiye were generally less likely to engage in educational activities with their mothers than those in the north (AOR = 0.46 95% CI: 0.23–0.91) (Table 5). In urban residences, fathers were 1.9 times more likely to engage in an educational activity with their child than in rural residencies (95% CI: 1.09–3.31) (Table 5). In households with less than five people, children were approximately two times more likely to engage in an activity with their mothers, fathers, or any adult (95% CI: 1.54–2.67; 1.33–2.84; 1.27–3.00, respectively) (Table 5). In households with a poorer wealth index, children were overall less likely to engage in educational activities with their mothers and fathers compared to richer households (AOR = 0.28 [95% CI: 0.19–0.42] and AOR = 0.55 [95% CI: 0.31–0.95], respectively) (Table 5).

Increased maternal age at birth increased the likelihood of engaging in activities with the father or any adult 2.64 times compared to mothers younger than 25 at the time of the birth (95% CI: 1.30–5.36 and 1.21–5.80, respectively) (Table 5). Increased maternal and paternal education also increased the likelihood of participating in an activity with any adult significantly (Table 5). Increased maternal education was associated with almost two times increased likelihood of engaging in an activity with any adult (both mother, father, and any adult) (Table 5). Increased paternal education has increased this likelihood approximately 1.5 times in engagement in an activity with mother and father (Table 5). Furthermore, children with working mothers had a higher probability of engaging in an activity with the father and an adult almost two times (AOR = 1.75 [95% CI: 1.21–2.54], OR = 1.92; [95% CI: 1.29–2.87]) (Table 5).

Also, in families with mothers who had UMNFP, fathers were less likely to engage in activities with the child (AOR = 0.30 [95% CI: 0.14–0.62]) or any adult (AOR = 0.35 [95% CI: 0.15–0.79]).

If a child was the fourth or later-born child in the family, there was a significant decrease in the likelihood of engaging in activities with the mother, father, or any adult. This decrease was statistically significant across all three groups when compared to the first-born child (Table 5). Also mothers, fathers, and adults were more likely to engage in activities with their children who were normal or large sized at birth (Table 5).

### 3.7. Further Analysis: Early Childhood Development Index Performance

Region, residence, household size, and wealth index were not associated with ECDI outcomes (Table 6).

Furthermore, the data suggest that a father aged 35 or older at birth increases the likelihood of being on track in socioemotional domain (AOR = 1.69, % 95 CI: 1.01–2.81), and in the total ECDI (AOR:2.63 [95% CI: 1.35–5.1]). Second and third born children regardless of the interval between siblings, are less likely to perform high scores in reading domain of the ECDI (AOR = 0.28 [95% CI: 0.11–0.70] AOR = 0.46 [95% CI: 0.30–0.71], respectively) (Table 6).

Female children are 1.47 times more likely to have better socio-emotional outcomes (95% CI: 1.09–1.98) and 1.43 times more likely to have better outcomes in the overall ECDI than male children (95% CI: 1.05–1.95) (Table 6). Older children also were 2 times more likely to be on-track in reading domain (95% CI: 1.44–3.14) and 1.38 times more likely to be on track in ECDI overall (%95 CI: 1.01–1.88). Additionally, the presence of three or more books at home increases the odds of being on-track 2.25 times in the literacy domain (%95 CI: 1.37–3.70) (Table 6). Also, participating in activities with the mother increased the likelihood of being on track in overall ECDI (AOR = 1.57 [%95 CI: 1.09–2.27]) and participating in activities with father elevated the possibility of being on track in both the socio-emotional domain and overall ECDI (AOR = 1.76 [%95 CI: 1.04–3.01] and AOR= 2.36 [%95 CI: 1.28–4.36], respectively).

## 4. Discussion

This study examined the impact of UMNFP and various socio-demographic factors on ECD outcomes in Türkiye, using data from the TDHS 2018. The results reveal important insights into the relationship between family structure, socioeconomic status, and ECD outcomes, particularly focusing on the ECDI. Contrary to our initial hypothesis, UMNFP was not significantly related to most caregiving practices or ECD scores. However, other socio-demographic factors such as maternal education, household income, and parental engagement activities emerged as critical determinants of children’s developmental success.

### 4.1. Unmet Family Planning Needs and Child Development

In our study, we found no relationship between UMNFP and ECDI. However, there is a strong relationship between the presence of UMNFP and engaging in educational activities with the father and adults. This situation could suggest a relationship where an increase in the number of children leads to a diminished distribution of attention [41]. This disparity not being observed among mothers can be explained by the cultural context in Turkey, where mothers are generally the primary caregivers. In Turkish society, one of the most significant responsibilities attributed to mothers is the care and upbringing of children [42] and fathers contribution to child care is usually limited to performing activities or transportation [43]. This cultural expectation likely influences how resources and responsibilities are allocated within families, potentially mitigating the impact of having more children on the mothers’ and fathers’ ability to provide care. As supported by our results above, engaging in activities with parents likely influences children’s ECDI outcomes. Consequently, it is plausible that UMNFP indirectly impacts child development through its influence on parent–child interactions. This finding also aligns with previous literature that suggests UMNFP and high maternal parity are linked to poorer child health [15,18,44]. One possible explanation is that while UMNFP may be related to maternal and neonatal health outcomes, these effects may not directly translate into variations in ECD as measured by the ECDI. Alternatively, the strong influence of other socioeconomic and household factors may have mitigated the potential negative impact of UMNFP on child development outcomes.

While we may not observe a direct effect of UMNFP on the determinants of ECD, we have shown that having a higher number of children impacts the frequency of activities engaged with them, which could highlight the importance of family planning. Additionally, we demonstrated that having less than five household members also related to the number of activities engaged with children, suggesting an indirect relationship with family planning. House crowding and chaos is also negatively related to learning behaviors and activities in the literature [45]. We identified that mothers who had their last child either unintentionally or not at their preferred time are more likely to expose their children to what is defined as inadequate care.

### 4.2. Socio-Demographic Factors Influencing Child Development

In our study, we did not find a significant effect of household income, number of household members, region, or type of residence on the overall ECDI score in the regression analysis. We believe this may be due to the small size of our study population. However, numerous studies in the literature have reported that these factors influence ECDI scores [46,47,48,49]. Higher levels of maternal education were associated with better outcomes in the literacy–numeracy, socio-emotional, and overall ECDI scores, which aligns with previous research that underscores the importance of maternal education in promoting child health and development [50]. Educated mothers may be more aware of the importance of early childhood stimulation, leading to greater engagement in learning-promoting activities such as reading and playing with educational toys. Moreover, the significant role of maternal education in the socio-emotional domain suggests that better-educated mothers may foster more supportive and emotionally responsive home environments [50,51].

A higher household income was also positively correlated with better ECDI scores, particularly in the literacy–numeracy domain. This finding is consistent with the literature, which shows that socioeconomic status plays a crucial role in access to educational materials and stimulating environments. Wealthier households were more likely to have books and toys that promote early learning, which are key resources for fostering cognitive development. Additionally, children from wealthier households were more likely to engage in multiple learning-promoting activities with parents, highlighting the resource-intensive nature of effective early childhood education.

Although the primary analysis showed that owning more than two types of toys influenced ECDI scores, this effect also disappeared in the regression analysis after adjusting for other confounding factors. This outcome could be due to the association between toy ownership and socioeconomic status. Children whose mothers have UMNFP are less likely to own three or more books, and this likelihood does not change even when adjusted for socioeconomic factors in the regression analysis. This indicates that the impact of UMNFP on book ownership is consistent despite socioeconomic considerations.

### 4.3. Household Structure and Parental Engagementk

Another significant finding was the role of the household structure in determining ECD outcomes. Children from smaller households (fewer than five members) performed better in literacy–numeracy and socio-emotional domains, suggesting that smaller family sizes may allow for more focused parental attention and resources per child. This is in line with previous research that suggests smaller family sizes are associated with better educational outcomes, as parents can invest more time and resources into each child [52]. However, this effect was also lost in the regression analysis, suggesting that there are some other factor that might be mediating these effects in our study.

Interestingly, paternal education also emerged as significant predictors of better child development outcomes, particularly in the literacy–numeracy and socio-emotional domains. This finding supports emerging evidence that fathers play an important role in ECD, particularly in families where fathers engage in caregiving activities [17,53,54,55]. The higher ECDI scores among children with employed mothers may also be attributed to the economic stability provided by dual-income households, which could enable greater access to educational resources. Although these effects were observed in the primary analysis, they disappeared in the multiple regression analysis when evaluated in terms of relationships with other variables. The presence of multiple variables influencing the issue of education and development, and the impossibility of evaluating all of them in this study, may have caused this situation. Therefore, there is a need for larger studies to investigate more variables related to early childhood education and development.

Moreover, parental engagement, particularly maternal and paternal involvement in learning-promoting activities, was found to be a key factor influencing ECD outcomes. Children who participated in four or more educational activities with their mothers or fathers or any adult in the three days prior to the survey were significantly more frequently on track in the literary–numerary domain, socio-emotional domain and overall ECDI. This finding highlights the critical role of responsive caregiving and parental involvement in fostering ECD. It is particularly notable that this relationship was present even after controlling for socioeconomic factors, suggesting that the quality of parental engagement may be as important as the material resources available to children.

### 4.4. Regional and Birth-Related Factors

Regional differences in child development outcomes were also observed, with children from the eastern regions of Türkiye being on track less frequently in literacy–numeracy and socio-emotional domains. These regional differences likely reflect broader socioeconomic disparities within the country [30], where children in wealthier regions may have better access to quality early childhood education and healthcare services.

In ECDI assessments in the literature, older children are more frequently evaluated as being on track. This trend is similarly observed in our study. Additionally, children who are third or fourth in the birth order have demonstrated better performance in reading, which can be explained by their older age. Likewise, consistent with the literature, female children have also shown better ECDI performance in both primary analysis and regression analysis [31,56,57].

### 4.5. Limitations

Although this study provides valuable insights into the factors influencing ECD in Türkiye, it is not without limitations. First, the cross-sectional nature of the TDHS data limits the ability to infer causality. Longitudinal studies are needed to examine how these factors influence child development trajectories over time. Second, the study relied on maternal self-reporting, which may introduce bias, particularly for variables such as the number of activities conducted with the child. Additionally, the relatively small number of children who did not achieve passing scores in certain domains limited the ability to conduct more detailed subgroup analyses in areas such as physical and learning development.

While the exclusions of multiple pregnancies, preterm births, and children not living with their mothers limit the generalizability of the findings to the broader population, this restriction ensures a more focused analysis of typical developmental trajectories. Consequently, the study provides more specific and relevant insights into the developmental outcomes of children under typical conditions, minimizing confounding factors and enhancing the precision of the findings. To address potential limitations stemming from the exclusion criteria and enhance representativeness, the study utilized CSPLAN files and weighting factors.

The study was conducted using the 2018 TDHS dataset because ECDI questions were included only in the 2018 survey. More comprehensive studies could be conducted by incorporating data from subsequent TDHS surveys. Also, the fact that the study relies on data from 2018 may raise concerns about the timeliness of the findings. However, it is important to note that no TDHS survey has been published in Turkey since 2018, which highlights the necessity for updated data. Future studies can address this limitation by including more recent data from ongoing or upcoming surveys.

While this study offers valuable insights within the Turkish context, the findings may not be generalizable to other countries with different socioeconomic and cultural contexts. Future research could expand on these findings by examining similar variables in diverse settings to understand how universal or culturally specific the relationships between family planning practices, childcare, and ECD may be.

Future research should explore the potential moderating role of cultural and regional differences in family planning practices and child development outcomes. Additionally, interventions aimed at promoting paternal involvement and supporting maternal education may have significant benefits for ECD, particularly in low- and middle-income countries. Furthermore, investigating the role of policy changes and healthcare access in influencing child development could provide more targeted interventions in both rural and urban populations.

## 5. Conclusions

In conclusion, this study highlights the significant role of household size, parental engagement and education, and regional disparities in shaping ECD outcomes in Türkiye. Although UMNFP did not emerge as a significant predictor of child development outcomes, the findings underscore the importance of socioeconomic factors and responsive caregiving practices. Policymakers should focus on promoting parental education, improving access to early childhood education resources, and encouraging parental involvement to support optimal child development outcomes.

## Figures and Tables

**Table 1:** Inadequate care, staying alone, staying with a young child, and educational items at home for children under 5 years of age according to variables *.

	Overall	Inadequate Care		Staying Alone		Staying with a Young Child		Having ≥2 Types of Playthings		Having ≥3 Books at Home	
		n (%)	*p*	n (%)	*p*	n (%)	*p*	n (%)	*p*	n (%)	*p*
**Total**	2390	158 (6.6)		81 (3.4)		100 (4.1)		1831 (76.6)		716 (29.9)	
**Region**			0.464		0.137		0.712		<0.001		<0.001
West	926	56 (6.0)		24 (2.6)		42 (4.5)		748 (80.8) ^a^		381 (41.1) ^a^	
South	328	27 (8.2)		16 (4.9)		14 (4.3)		247 (75.3) ^b^		57 (17.4) ^b^	
Middle	430	29 (6.7)		19 (4.4)		13 (3.0)		351 (81.6) ^a^		166 (38.6) ^a^	
North	90	3 (3.3)		1 (1.1)		3 (3.3)		74 (81.3) ^ab^		35 (38.5) ^a^	
East	616	43 (7.0)		20 (3.2)		28 (4.6)		411 (66.7) ^c^		77 (12.5) ^c^	
**Residence**			0.034		0.065		0.080		<0.001		<0.001
Urban	1802	108 (6.0)		54 (3.0)		68 (3.8)		1414 (78.5)		621 (34.5)	
Rural	588	50 (8.5)		27 (4.6)		32 (5.4)		417 (70.8)		93 (15.8)	
**Household size**			<0.001		0.912		<0.001		<0.001		<0.001
<5 person	1061	49 (4.6)		36 (3.4)		23 (2.2)		862 (81.2)		457 (43.1)	
≥5 person	1329	110 (8.3)		44 (3.3)		78 (5.9)		970 (73.0)		257 (19.4)	
**Wealth index**			0.021		0.126		0.221		<0.001		<0.001
Poorer	1042	83 (8.0) ^a^		44 (4.2)		50 (4.8)		711 (68.2) ^a^		103 (9.9) ^a^	
Middle	492	33 (6.7) ^a.b^		15 (3.0)		23 (4.7)		408 (82.9) ^b^		132 (26.8) ^b^	
Richer	856	41 (4.8) ^b^		22 (2.6)		28 (3.3)		712 (83.2) ^b^		479 (56.0) ^c^	
**Mother’s age at birth**			0.593		0.280		0.069		0.092		<0.001
<25 years	720	42 (5.8)		30 (4.2)		20 (2.8)		533 (74.0)		162 (22.5) ^a^	
25–34 years	1305	90 (6.9)		41 (3.1)		63 (4.8)		1022 (78.3)		443 (33.9) ^b^	
≥35 years	364	26 (7.1)		9 (2.5)		18 (4.9)		276 (75.8)		110 (30.1) ^b^	
**Father’s age at birth**			0.798		0.471		0.143		0.518		0.012
<25 years	225	13 (5.8)		8 (3.5)		8 (3.6)		166 (73.8)		48 (21.3) ^a^	
25–34 years	1337	88 (6.6)		50 (3.7)		49 (3.7)		1033 (77.3)		415 (31.0) ^b^	
≥35 years	799	56 (7.0)		22 (2.8)		43 (5.4)		613 (76.6)		245 (30.6) ^b^	
**Mother’s Education**			0.100		0.217		0.008		<0.001		<0.001
<Secondary	1135	85 (7.5)		33 (2.9)		61 (5.4)		828 (73.0)		177 (15.6)	
≥Secondary	1255	73 (5.8)		48 (3.8)		40 (3.2)		1004 (79.9)		537 (42.8)	
**Father’s Education**			<0.001		0.557		<0.001		0.001		<0.001
<Secondary	676	68 (10.1)		25 (3.7)		48 (7.1)		486 (72.0)		95 (14.1)	
≥Secondary	1707	91 (5.3)		55 (3.2)		52 (3.0)		1340 (78.5)		619 (36.3)	
**Mother’s working status**			0.702		0.948		0.592		0.001		<0.001
Not working	1919	125 (6.5)		64 (3.3)		79 (4.1)		1447 (75.1)		476 (24.8)	
Working	471	33 (7.0)		16 (3.4)		22 (4.7)		389 (82.6)		239 (50.6)	
**Mother currently pregnant**			0.055		0.814		0.005		0.286		0.349
No or unsure	2224	153 (6.9)		75 (3.4)		101 (4.5)		1699 (76.4)		670 (30.1)	
Yes	166	5 (3.0)		5 (3.0)		0 (0.0)		132 (80.0)		44 (26.7)	
**Wanted last child**			0.004		0.696		<0.001		0.923		0.002
Wanted then	1637	105 (6.4) ^b^		56 (3.4)		62 (3.8) ^a^		1253 (76.5)		510 (31.2) ^a^	
Wanted later	302	10 (3.3) ^a^		8 (2.6)		6 (2.0) ^a^		234 (77.5)		100 (33.1) ^a^	
Wanted no more	451	42 (9.3) ^c^		17 (3.8)		33 (7.3) ^b^		344 (76.3)		105 (23.3) ^b^	
**Unmet FP need**			0.979		0.152		0.057		0.013		<0.001
No	2074	137 (6.6)		66 (3.2)		94 (4.5)		1607 (77.5)		665 (32.1)	
Yes	316	21 (6.6)		15 (4.7)		7 (2.2)		224 (71.1)		49 (15.6)	
**Number of mother’s birth**			<0.001		0.276		<0.001		0.001		<0.001
1	561	17 (3.0) ^a^		14 (2.5)		10 (1.8) ^a^		437 (77.9) ^a^		242 (43.1) ^a^	
2–3	1410	100 (7.0) ^b^		54 (3.8)		58 (4.1) ^b^		1102 (78.2^) a^		431 (30.6) ^b^	
≥4	419	42 (10.0) ^c^		12 (2.9)		33 (7.9) ^c^		292 (69.7) ^b^		41 (9.8) ^c^	
**Birth order**			<0.001		0.888		<0.001		<0.001		<0.001
Firstborn	783	31 (4.0) ^a^		26 (3.3)		15 (1.9) ^a^		626 (79.8) ^a^		310 (39.5) ^a^	
2–3rd born after <24 mo	255	17 (6.7) ^a,b^		9 (3.5)		11 (4.3) ^b^		180 (70.6) ^b^		46 (18.0) ^c^	
2–3rd born after ≥24 mo	994	72 (7.2) ^b^		35 (3.5)		45 (4.5) ^b^		785 (79.0) ^b^		330 (33.2) ^b^	
≥4th born after <24 mo	76	5 (6.6) ^a,b,c^		1 (1.3)		5 (6.6) ^b,c^		48 (63.2) ^b,c^		10 (13.2) ^cd^	
≥4th born after ≥24 mo	281	33 (11.7) ^c^		10 (3.6)		25 (8.9) ^c^		192 (68.6) ^c^		19 (6.8) ^d^	
**Gestational duration**			0.147		0.044		0.792		0.875		0.675
32–36 week	403	20 (5.0)		7 (1.7)		18 (4.5)		310 (76.9)		117 (29.0)	
≥37 week	1987	138 (6.9)		74 (3.7)		83 (4.2)		1522 (76.6)		598 (30.1)	
**Size at birth**			0.948		0.106		0.914		0.042		0.001
Large	326	20 (6.1)		6 (1.8)		14 (4.3)		262 (80.4) ^a^		125 (38.3) ^a^	
Normal	1570	104 (6.6)		50 (3.2)		68 (4.3)		1208 (77.0) ^a^		461 (29.4) ^b^	
Small	488	32 (6.6)		22 (4.5)		19 (3.9)		356 (73.0) ^b^		129 (26.4) ^b^	
**Child age**			0.047		0.589		0.034		<0.001		<0.001
0 years old	479	19 (4.0) ^a^		12 (2.5)		11 (2.3) ^a^		176 (36.7) ^a^		31 (6.5) ^a^	
1 year old	430	27 (6.3) ^a,b^		16 (3.7)		14 (3.2) ^a,b^		361 (84.0) ^b^		71 (16.5) ^b^	
2 years old	467	33 (7.1) ^b^		15 (3.2)		25 (5.3) ^b^		417 (89.3) ^c^		159 (34.0) ^c^	
3 years old	487	43 (8.8) ^b^		21 (4.3)		29 (6.0) ^b^		423 (86.7) ^b,c^		200 (41.1) ^d^	
4 years old	526	37 (7.0) ^b^		16 (3.0)		23 (4.4) ^a,b^		454 (86.3) ^b,c^		254 (48.3) ^e^	
**Child sex**			0.160		0.101		0.172		0.836		0.203
Male	1188	70 (5.9)		33 (2.8)		43 (3.6)		908 (76.4)		341 (28.7)	
Female	1202	88 (7.3)		48 (4.0)		57 (4.7)		923 (76.8)		374 (31.1)	

* Data are present for children under 5 years of age, n (%, row percentages). The letters ^a,b,c,d,e^ were used to indicate the difference between the groups in the same column for the selected variable. FP, family planning; BMI, body mass index.

**Table 2:** Factors associated with activity engagement status of the children aged 24–59 months in the last 3 days preceding the survey *.

	Overall	Engaging in ≥4 Activities with Mother		Engaging in ≥4 Activities with Father		Engaging in ≥4 Activities with an Adult Household Member (Aged 15 or Older)	
		n (%)	*p*	n (%)	*p*	n (%)	*p*
**Total**	1480	738 (49.8)		233 (15.7)		979 (66.0)	
**Region**			<0.001		<0.001		<0.001
West	587	362 (61.8) ^a^		122 (20.8) ^a^		442 (75.3) ^a^	
South	140	88 (46.6) ^b^		28 (14.7) ^a^		140 (74.1) ^a^	
Middle	177	141 (51.3) ^b^		57 (20.7) ^a^		177 (64.1) ^b^	
North	43	34 (60.7) ^a.b^		8 (14.3) ^a^		43 (76.8) ^a.b^	
East	178	113 (30.3) ^c^		18 (4.8) ^b^		178 (47.7) ^c^	
**Residence**			<0.001		<0.001		<0.001
Urban	1121	613 (54.6)		210 (18.7)		788 (70.3)	
Rural	359	127 (35.4)		23 (6.4)		191 (53.2)	
**Household size**			<0.001		<0.001		<0.001
<5	659	457 (69.2)		178 (27.0)		528 (80.1)	
≥5	821	282 (34.3)		55 (6.7)		451 (54.9)	
**Wealth index**			<0.001		<0.001		<0.001
Poorer	641	180 (28.1) ^a^		33 (5.1) ^a^		306 (47.7) ^a^	
Middle	309	171 (55.2) ^b^		47 (15.2) ^b^		225 (72.8) ^b^	
Richer	530	389 (73.4) ^c^		153 (28.9) ^c^		448 (84.5) ^c^	
**Mother’s age at birth**			0.327		0.236		0.014
<25 years	452	214 (47.3)		60 (13.3)		275 (60.8) ^a^	
25–34 years	823	425 (51.6)		139 (16.9)		567 (68.9) ^b^	
≥35 years	206	101 (49.0)		33 (16.0)		138 (67.0) ^a.b^	
**Father’s age at birth**			0.099		0.046		0.185
<25 years	145	64 (44.4)		18 (12.5) ^a.b^		86 (59.3)	
25–34 years	830	434 (52.3)		150 (18.1) ^b^		554 (66.7)	
≥35 years	481	229 (47.6)		65 (13.5) ^a^		323 (67.2)	
**Mother’s Education**			<0.001		<0.001		<0.001
<Secondary	742	243 (32.8)		47 (6.3)		387 (52.2)	
≥Secondary	739	496 (67.1)		186 (25.2)		593 (80.2)	
**Father’s Education**			<0.001		<0.001		<0.001
<Secondary	1023	599 (58.6)		211 (20.6)		754 (73.7)	
≥Secondary	453	139 (30.7)		22 (4.9)		224 (49.4)	
**Maternal current working status**			<0.001		<0.001		0.002
Not working	1176	557 (47.4)		150 (12.8)		755 (64.2)	
Working	304	182 (59.9)		83 (27.2)		224 (73.7)	
**Mother currently pregnant**			0.188		0.323		0.027
No or unsure	1359	686 (50.5)		210 (15.5)		910 (67.0)	
Yes	121	54 (44.3)		23 (18.9)		69 (57.0)	
**Wanted last child**			<0.001		<0.001		0.116
Wanted then	978	518 (53.0) ^a^		182 (18.6) ^a^		661 (67.6)	
Wanted later	188	95 (50.5) ^a^		39 (20.6) ^a^		126 (67.0)	
Wanted no more	315	126 (40.1) ^b^		13 (4.1) ^b^		193 (61.3)	
**Unmet FP need**			0.001		<0.001		<0.001
No	1308	674 (51.6)		224 (17.1)		887 (67.8)	
Yes	173	65 (37.6)		9 (5.2)		93 (53.8)	
**Number of mother’s birth**			<0.001		<0.001		<0.001
1	281	222 (78.7) ^a^		102 (36.3) ^a^		241 (85.8) ^a^	
2–3	905	454 (50.2) ^b^		127 (14.0) ^b^		602 (66.5) ^b^	
≥4	294	63 (21.5) ^c^		4 (1.4) ^c^		136 (46.3) ^c^	
**Birth order**			<0.001		<0.001		<0.001
Firstborn	485	321 (66.2) ^a^		136 (28.0) ^a^		374 (38.2) ^a^	
2–3rd born after <24 mo	155	65 (41.7) ^b^		15 (9.6) ^b.c^		87 (8.9) ^b^	
2–3rd born after ≥24 mo	604	303 (50.2) ^b^		79 (13.1) ^c^		406 (41.5) ^c^	
≥4th born after <24 mo	56	5 (8.9) ^c^		1 (1.8) ^b.d^		24 (2.5) ^b^	
≥4th born after ≥24 mo	179	45 (25.1) ^d^		3 (1.7) ^d^		87 (8.9) ^b^	
**Gestational duration**			0.354		0.075		0.419
32–36 week	264	125 (47.3)		32 (12.1)		169 (64.0)	
≥37 week	1216	614 (50.5)		201 (16.5)		810 (66.6)	
**Size at birth**			0.005		0.010		0.057
Large	235	127 (53.8) ^a^		32 (13.6) ^a^		165 (70.2)	
Normal	931	481 (51.7) ^a^		167 (17.9) ^a^		623 (66.9)	
Small	307	129 (41.9) ^b^		34 (11.1) ^b^		187 (60.9)	
**Child age**			0.807		0.489		0.639
2 years old	467	231 (49.5)		81 (17.3)		308 (66.0)	
3 years old	487	240 (49.3)		75 (15.4)		315 (64.7)	
4 years old	526	269 (51.1)		77 (14.6)		355 (67.5)	
**Child sex**			0.857		0.167		0.488
Male	766	384 (50.1)		111 (14.5)		513 (67.0)	
Female	714	355 (49.7)		122 (17.1)		466 (65.3)	

* Data are present for children between 24 and 60 years of age, %: row percentages. The letters ^a,b,c,d^ were used to indicate the difference between the groups in the same column for the selected variable. BMI, body mass index; FP, family planning.

**Table 3:** Early childhood index (ECDI) and some subdomains according to study variables.

	Overall	ECDI Literacy–Numeracy		ECDI Socio-Emotional		ECDI Total	
		n (%)	*p*	n (%)	*p*	n (%)	*p*
**Total**	1013	148 (14.6)		749 (74.0)		752 (74.3)	
**Region**			<0.001		0.137		0.008
West	402	74 (18.4) ^a^		308 (76.6)		317 (78.9) ^a^	
South	128	16 (12.5) ^a^		98 (76.6)		97 (75.2) ^a.b^	
Middle	183	37 (20.2) ^a^		137 (74.9)		139 (76.0) ^a^	
North	40	6 (15.0) ^a^		25 (62.5)		27 (67.5) ^a.b^	
East	260	15 (5.8) ^b^		181 (69.9)		173 (66.5) ^b^	
**Residence**			0.002		0.826		0.588
Urban	760	126 (16.6)		564 (74.1)		568 (74.7)	
Rural	252	22 (8.7)		185 (73.4)		184 (73.0)	
**Household size**			0.010		0.003		<0.001
<5 person	442	79 (17.9)		349 (78.6)		353 (79.7)	
≥5 person	570	69 (12.1)		401 (70.4)		399 (70.0)	
**Wealth index**			<0.001		<0.001		<0.001
Poorer	450	39 (8.7) ^a^		303 (67.0) ^a^		297 (65.9) ^a^	
Middle	210	33 (15.7) ^b^		170 (80.6) ^b^		170 (80.6) ^b^	
Richer	351	75 (21.4) ^b^		277 (78.9) ^b^		286 (81.5) ^b^	
**Mother’s age at birth**			0.802		0.555		0.173
<25 years	313	45 (14.4)		229 (73.2)		227 (72.5)	
25–34 years	562	85 (15.1)		413 (73.5)		413 (73.6)	
≥35 years	139	18 (12.9)		108 (77.7)		112 (80.6)	
**Father’s age at birth**			0.648		0.021		0.003
<25 years	105	15 (14.3)		67 (63.8) ^a^		68 (64.8) ^a^	
25–34 years	555	78 (14.1)		412 (74.2) ^b^		407 (73.3) ^a^	
≥35 years	331	54 (16.3)		257 (77.4) ^b^		266 (80.4) ^b^	
**Mother’s Education**			0.001		<0.001		<0.001
<Secondary	522	58 (11.1)		362 (69.2)		356 (68.1)	
≥Secondary	489	89 (18.2)		388 (79.0)		396 (80.8)	
**Father’s Education**			0.005		0.096		0.002
<Secondary	326	33 (10.1)		231 (70.6)		222 (68.1)	
≥Secondary	683	115 (16.8)		516 (75.5)		527 (77.2)	
**Maternal current working status**			0.477		0.013		0.017
Not working	787	111 (14.1)		568 (72.2)		571 (72.6)	
Working	225	36 (16.0)		181 (80.4)		181 (80.4)	
**Wanted last child**			0.006		0.083		0.342
Wanted then	672	113 (16.8) ^a^		506 (75.2)		505 (74.9)	
Wanted later	122	8 (6.6) ^b^		94 (77.7)		94 (77.0)	
Wanted no more	217	26 (12.0) ^a.b^		149 (68.3)		154 (70.6)	
**Unmet FP need**			0.696		0.633		0.838
No	892	131 (14.7)		659 (73.8)		662 (74.1)	
Yes	120	16 (13.3)		91 (75.8)		90 (75.0)	
**Mother currently pregnant**			0.893		0.073		0.062
No or unsure	927	136 (14.7)		694 (74.8)		697 (75.1)	
Yes	85	12 (14.1)		56 (65.9)		56 (65.9)	
**Number of mother’s birth**			<0.001		0.092		0.065
1	169	43 (25.4) ^a^		134 (79.3)		134 (79.9)	
2–3	642	89 (13.9) ^b^		475 (74.0)		480 (74.8)	
≥4	202	16 (7.9) ^c^		140 (69.3)		139 (68.8)	
**Birth order**			<0.001		0.300		0.164
Firstborn	325	75 (23.1) ^a^		248 (76.3)		251 (77.2)	
2–3rd born after <24 mo	102	6 (5.9) ^b^		70 (69.3)		69 (68.3)	
2–3rd born after ≥24 mo	437	54 (12.4) ^b^		328 (75.1)		328 (75.1)	
4th or more born after <24 mo	34	2 (5.9) ^b^		22 (64.7)		21 (61.8)	
4th or more born after ≥24 mo	115	10 (8.7) ^b^		81 (69.8)		84 (72.4)	
**Gestational duration**			0.190		0.582		0.512
32–36 weeks	191	22 (11.5)		139 (72.4)		139 (72.4)	
≥37 weeks	820	125 (15.2)		611 (74.3)		614 (74.7)	
**Size at birth**			0.296		0.955		0.274
Large	159	22 (13.8)		117 (73.1)		112 (69.6)	
Normal	634	101 (15.9)		471 (74.3)		480 (75.7)	
Small	214	25 (11.7)		158 (74.2)		157 (73.7)	
**Child age**			<0.001		0.467		0.018
3 years	487	48 (9.9)		355 (72.9)		345 (70.8)	
4 years	525	100 (19.0)		394 (74.9)		407 (77.4)	
**Child sex**			0.961		0.004		0.005
Male	532	77 (14.5)		374 (70.3)		376 (70.7)	
Female	481	70 (14.6)		376 (78.2)		377 (78.4)	
**Presence of ≥3 children’s books**			<0.001		0.001		<0.001
No	559	43 (7.7)		391 (69.9)		381 (68.2)	
Yes	454	105 (23.1)		358 (78.9)		371 (81.7)	
**Presence of ≥2 types of toys**			0.298		0.081		0.002
No	137	16 (11.7)		93 (67.9)		86 (63.2)	
Yes	877	132 (15.1)		657 (74.9)		666 (75.9)	
**Insufficient care**			0.857		0.239		0.150
Sufficient	934	137 (14.7)		695 (74.4)		699 (74.8)	
Insufficient	79	11 (13.9)		54 (68.4)		54 (67.5)	
**≥4 activities with mother**			<0.001		<0.001		<0.001
No	505	44 (8.7)		349 (69.1)		338 (66.9)	
Yes	508	104 (20.5)		400 (78.7)		415 (81.5)	
**≥4 activities with father**			<0.001		<0.001		<0.001
No	892	110 (12.8)		618 (71.8)		615 (71.4)	
Yes	122	38 (25.0)		131 (86.2)		137 (90.1)	
**≥4 activities with an adult**			<0.001		0.004		<0.001
No	343	22 (6.4)		234 (68.4)		217 (63.5)	
Yes	670	126 (18.8)		515 (76.8)		535 (79.7)	

Data are present for children between 36 and 60 years of age; %: row percentages. The letters ^a,b,c^ were used to indicate the difference between the groups in the same column for the selected variable. FP, family planning.

**Table 4:** Logistic regression analysis of the factors effecting insufficient care and educational materials.

	Insufficient Care		Presence of ≥2 Types of Playthings		Presence of ≥3 Children’s Books	
	AOR	95% CI	AOR	95% CI	AOR	95% CI
**Region**						
West			0.90	(0.46–1.74)	1.00	(0.56–1.77)
South			0.90	(0.45–1.82)	0.58	(0.30–1.11)
Middle			0.86	(0.43–1.71)	0.69	(0.38–1.27)
North			1.00		1.00	
East			0.55	(0.28–1.08)	**0.48**	**(0.26–0.90)**
**Residence**						
Urban	0.86	(0.58–1.29)	1.01	(0.76–1.33)	1.09	(0.77–1.55)
Rural	1.00		1.00		1.00	
**Household size**						
<5 person	0.73	(0.49–1.10)	1.12	(0.85–1.47)	**1.42**	**(1.09–1.86)**
≥5 person	1.00		1.00		1.00	
**Wealth index**						
Poorer	1.24	(0.76–2.01)	**0.58**	**(0.40–0.83)**	**0.16**	**(0.11–0.23)**
Middle	1.21	(0.74–1.98)	1.41	(0.98–2.04)	**0.34**	**(0.25–0.46)**
Richer	1.00		1.00		1.00	
**Mother’s age at birth**						
<25 years			1.00		1.00	
25–34 years			1.24	(0.94–1.65)	**1.72**	**(1.25–2.36)**
≥35 years			1.51	(1.00–2.29)	**1.81**	**(1.09–2.99)**
**Father’s age at birth**						
<25 years					1.00	
25–34 years					0.88	(0.55–1.42)
≥35 years					0.97	(0.55–1.72)
**Mother’s Education**						
<Secondary	1.00		1.00		1.00	
≥Secondary	1.24	(0.84–1.83)	1.05	(0.80–1.37)	**2.24**	**(1.70–2.96)**
**Father’s Education**						
<Secondary	1.00		1.00		1.00	
≥Secondary	0.63	(0.43–0.92)	1.24	(0.95–1.63)	**1.66**	**(1.21–2.28)**
**Maternal current working status**						
Not working			1.00		1.00	
Working			1.34	(0.97–1.83)	**2.23**	**(1.67–2.99)**
**Mother currently pregnant**						
No or unsure	1.00					
Yes	0.4	(0.16–1.02)	1.06	(0.66–1.70)		
**Unmet FP need**						
No	1.00		1.00		1.00	
Yes	1.16	(0.7–1.93)	0.86	(0.61–1.19)	**0.52**	**(0.35–0.78)**
**Birth order**						
Firstborn	1.00		1.00		1.00	
2–3rd born after <24 mo	1.37	(0.73–2.59)	0.85	(0.57–1.27)	**0.60**	**(0.38–0.94)**
2–3rd born after ≥24 mo	**1.57**	**(1.00–2.46)**	0.92	(0.68–1.24)	**0.74**	**(0.55–1.00)**
4th or more born after <24 mo	1.19	(0.43–3.30)	**0.45**	**(0.24–0.84)**	0.53	(0.21–1.32)
4th or more born after ≥24 mo	**2.15**	**(1.19–3.91)**	**0.59**	**(0.37–0.92)**	**0.25**	**(0.13–0.49)**
**Gestational duration**						
32–36 weeks	0.66	(0.41–1.08)				
≥37 weeks	1.00					
**Size at birth**						
Large			1.18	(0.79–1.76)	**1.60**	**(1.07–2.41)**
Normal			1.12	(0.85–1.48)	1.06	(0.78–1.45)
Small			1.00		1.00	
**Child age**						
0 year	1.00		1.00		1.00	
1 year	1.6	(0.87–2.93)	**11.8**	**(8.37–16.64)**	**3.75**	**(2.27–6.19)**
2 year	**1.88**	**(1.05–3.37)**	**18.96**	**(13.05–27.54)**	**14.25**	**(8.80–23.10)**
3 year	**2.40**	**(1.37–4.21)**	**15.80**	**(11.08–22.51)**	**29.90**	**(18.25–48.98)**
4 year	**1.85**	**(1.04–3.27)**	**13.98**	**(9.98–19.58)**	**41.56**	**(25.46–67.85)**
**Child sex**						
Male	1.00					
Female	1.29	(0.93–1.8)				

FP, family planning; AOR, adjusted odds ratio; CI, confidence interval.

**Table 5:** Logistic regression analysis of the factors effecting engaging with activities with an adult or a parent.

	≥4 Activities with Mother		≥4 Activities with Father		≥4 Activities with Any Adult	
	AOR	95% CI	AOR	95% CI	AOR	95% CI
**Region**						
West	0.81	(0.42–1.56)	1.10	(0.47–2.62)	1.25	(0.47–3.33)
South	0.83	(0.41–1.68)	1.26	(0.48–3.27)	1.31	(0.44–3.87)
Middle	**0.46**	**(0.23–0.91)**	1.10	(0.45–2.69)	1.28	(0.47–3.52)
North	1.00		1.00		1.00	
East	0.58	(0.29–1.13)	0.51	(0.19–1.35)	0.58	(0.19–1.77)
**Residence**						
Urban	0.86	(0.62–1.20)	**1.90**	**(1.09–3.31)**	1.52	(0.82–2.81)
Rural	1.00		1.00		1.00	
**Household size**						
<5 person	**2.03**	**(1.54–2.67)**	**1.94**	**(1.33–2.84)**	**1.95**	**(1.27–3.00)**
≥5 person	1.00		1.00		1.00	
**Wealth index**						
Poorer	**0.28**	**(0.19–0.42)**	**0.55**	**(0.31–0.95)**	0.53	(0.28–1.01)
Middle	**0.63**	**(0.45–0.89)**	0.75	(0.50–1.15)	0.68	(0.42–1.10)
Richer	1.00		1.00		1.00	
**Mother’s age at birth**						
<25 years	1.00		1.00		1.00	
25–34 years	1.36	(0.98–1.88)	**1.56**	**(1.01–2.40)**	1.57	(0.97–2.54)
≥35 years	1.51	(0.90–2.54)	**2.64**	**(1.30–5.36)**	**2.64**	**(1.21–5.80)**
**Father’s age at birth**						
<25 years	1.00		1.00		1.00	
25–34 years	1.11	(0.70–1.75)	1.17	(0.62–2.22)	1.07	(0.52–2.18)
≥35 years	1.14	(0.65–2.00)	1.00	(0.46–2.17)	1.11	(0.47–2.63)
**Mother’s Education**						
<Secondary	1.00		1.00		1.00	
≥secondary	**2.04**	**(1.55–2.69)**	**1.79**	**(1.19–2.67)**	**2.23**	**(1.39–3.58)**
**Father’s Education**						
<Secondary	1.00		1.00		1.00	
≥secondary	**1.50**	**(1.12–2.01)**	**1.79**	**(1.06–3.01)**	1.65	(0.90–3.04)
**Maternal working status**						
Not working	1.00		1.00		1.00	
Working	1.12	(0.81–1.55)	**1.75**	**(1.21–2.54)**	**1.92**	**(1.29–2.87)**
**Mother currently pregnant**						
No or unsure	1.00		1.00		1.00	
Yes	1.03	(0.63–1.69)	1.66	(0.89–3.08)	1.87	(0.98–3.59)
**Unmet FP need**						
No	1.00		1.00		1.00	
Yes	0.72	(0.48–1.07)	**0.30**	**(0.14–0.62)**	**0.35**	**(0.15–0.79)**
**Birth order**						
Firstborn	1.00		1.00		1.00	
2–3rd born after <24 mo	0.65	(0.42–1.01)	**0.48**	**(0.25–0.89)**	**0.41**	**(0.19–0.86)**
2–3rd born after ≥24 mo	**0.54**	**(0.39–0.75)**	**0.38**	**(0.26–0.57)**	**0.36**	**(0.23–0.55)**
4th or more born after <24 mo	**0.11**	**(0.04–0.31)**	0.14	(0.02–1.13)	0.06	(0.00–1.82)
4th or more born after ≥24 mo	**0.50**	**(0.30–0.85)**	**0.13**	**(0.03–0.48)**	**0.08**	**(0.01–0.52)**
**Gestational duration**						
32–36 weeks	0.91	(0.66–1.26)	1.19	(0.75–1.9)	1.29	(0.76–2.18)
≥37 weeks	1.00		1.00		1.00	
**Size at birth**						
Large	**1.71**	**(1.13–2.59)**	1.16	(0.65–2.07)	1.52	(0.8–2.89)
Normal	1.29	(0.94–1.77)	**1.60**	**(1.02–2.5)**	**1.66**	**(1.00–2.77)**
Small	1.00		1.00		1.00	
**Child sex**						
Male			1.00			
Female			1.17	(0.85–1.61)		

FP, family planning; AOR, adjusted odds ratio; CI, confidence interval.

**Table 6:** Logistic regression analysis of the factors associated with early childhood index score and some subdomains.

	ECDI Literacy–Numeracy		ECDI Socioemotional		ECDI Total	
	AOR	95% CI	AOR	95% CI	AOR	95% CI
**Region**						
West	1.20	(0.46–3.09)	1.91	(0.91–4.00)	1.88	(0.87–4.08)
South	1.03	(0.35–3.03)	2.56	(1.12–5.84)	2.18	(0.93–5.13)
Middle	1.33	(0.5–3.59)	1.63	(0.75–3.55)	1.55	(0.69–3.49)
North	1.00		1.00		1.00	
East	0.52	(0.18–1.51)	2.03	(0.95–4.35)	1.65	(0.75–3.64)
**Residence**						
Urban	1.55	(0.87–2.75)				
Rural	1.00					
**Household size**						
<5 person	0.70	(0.45–1.07)	1.19	(0.85–1.67)	1.05	(0.72–1.51)
≥5 person	1.00		1.00		1.00	
**Wealth index**						
Poorer	0.92	(0.50–1.70)	0.82	(0.52–1.28)	0.84	(0.52–1.34)
Middle	0.99	(0.60–1.66)	1.34	(0.84–2.14)	1.19	(0.73–1.94)
Richer	1.00		1.00		1.00	
**Mother’s age at birth**						
<25 years					1.00	
25–34 years					0.78	(0.52–1.18)
≥35 years					0.74	(0.38–1.45)
**Father’s age at birth**						
<25 years			1.00		1.00	
25–34 years			1.35	(0.84–2.15)	1.45	(0.85–2.48)
≥35 years			**1.69**	**(1.01–2.81)**	**2.63**	**(1.35–5.1)**
**Mother’s education**						
<Secondary	1.00		1.00		1.00	
≥Secondary	0.90	(0.58–1.4)	1.40	(0.99–1.99)	1.42	(0.99–2.05)
**Father’s education**						
<Secondary	1.00		1.00		1.00	
≥Secondary	1.11	(0.68–1.81)	0.95	(0.67–1.35)	1.20	(0.84–1.71)
**Maternal current working status**						
Not working			1.00		1.00	
Working			1.41	(0.95–2.1)	1.25	(0.83–1.9)
**Mother currently pregnant**						
No or unsure			1.00		1.00	
Yes			0.59	(0.34–1.00)	0.62	(0.35–1.08)
**Birth order**						
Firstborn	1.00				1.00	
2–3rd born after <24 mo	**0.28**	**(0.11–0.70)**			0.83	(0.48–1.46)
2–3rd born after ≥24 mo	**0.46**	**(0.30–0.71)**			0.84	(0.55–1.29)
4th or more after< 24 mo	0.56	(0.12–2.48)			0.80	(0.33–1.95)
4th or more after ≥24 mo	0.70	(0.31–1.55)			1.14	(0.59–2.20)
**Unmet family planning need**						
No	1.00		1.00		1.00	
Yes	1.13	(0.61–2.09)	1.56	(0.95–2.56)	1.59	(0.95–2.66)
**Gestational duration**						
32–36 weeks	0.82	(0.49–1.37)				
≥37 weeks	1.00					
**Child age**						
3 y	1.00				1.00	
4 y	**2.12**	**(1.44–3.14)**			**1.38**	**(1.01–1.88)**
**Child sex**						
Male			1.00		1.00	
Female			**1.47**	**(1.09–1.98)**	**1.43**	**(1.05–1.95)**
≥**Three books at home**						
Yes	**2.25**	**(1.37–3.70)**	0.95	(0.64–1.42)	1.04	(0.69–1.58)
No	1.00		1.00		1.00	
≥**Two types of toys at home**						
Yes			1.01	(0.66–1.56)	1.21	(0.78–1.87)
No			1.00		1.00	
**Insufficient care**						
Sufficient			1.24	(0.74–2.09)	1.19	(0.7–2.01)
Insufficient			1.00		1.00	
≥**Four activities with mom**						
Yes	1.50	(0.95–2.37)	1.31	(0.92–1.87)	**1.57**	**(1.09–2.27)**
No	1.00		1.00		1.00	
≥**Four activities with dad**						
Yes	1.15	(0.71–1.85)	**1.76**	**(1.04–3.01)**	**2.36**	**(1.28–4.36)**
No	1.00		1.00		1.00	

ECD, early childhood development; AOR, adjusted odds ratio; CI, confidence interval.

## Data Availability

Due to privacy, the data presented in this study are available on request from the corresponding author.
